# Case report: An occult hepatitis B virus infection reactivation in an HIV/HCV coinfected patient during an immune reconstitution inflammatory syndrome

**DOI:** 10.3389/fcimb.2023.1143346

**Published:** 2023-04-14

**Authors:** Serena Zaltron, Anna Cambianica, Marco Di Gregorio, Cosimo Colangelo, Samuele Storti, Giorgio Tiecco, Francesco Castelli, Eugenia Quiros-Roldan

**Affiliations:** ^1^ Unit of Infectious and Tropical Diseases, ASST Spedali Civili di Brescia, Brescia, Italy; ^2^ Unit of Infectious and Tropical Diseases, Department of Clinical and Experimental Sciences, ASST Spedali Civili di Brescia and University of Brescia, Brescia, Italy

**Keywords:** occult HBV, reactivation, HBV/HIV coinfection, immune reconstitution inflammatory syndrome (IRIS), hepatitis B virus

## Abstract

The natural history of occult hepatitis B virus infection (OBI) and the mechanism involved in HBV reactivation are only partially understood. As regards people living with HIV (PLWH), HBV reactivation is estimated to occur with an incidence ratio of 0.019 cases per 100 person-year. Here we report the case of OBI reactivation in a HIV/HCV co-infected patient followed for 25 years at our Infectious Diseases Unit, but, unfortunately, lost to follow-up about 19 months after Direct-acting antivirals (DAAs) treatment. At re-engagement, blood tests showed high replication of plasmatic HIV-RNA along with severe immunosuppression and normal levels of liver enzymes. However, 3 months after ART reintroduction, an immune reconstitution inflammatory syndrome (IRIS) was diagnosed with high detectable HBV-DNA load and transaminase elevation. Our case report shows how the balance between the virus and the host immune system is quite a dynamic process that might significantly impact the course of the disease. The aim of this case report is to bring to the attention of physicians that, although OBI reactivation is a rather rare occurrence, even amongst PLWH, its potential consequences compel to a high alertness on the matter. Therefore, especially in patients with an impaired immune system and on a tenofovir or lamivudine-sparing regimen, HBV serological and virological markers should always be strictly monitored, even in the absence of a hepatitis flare.

## Introduction

Hepatitis B virus (HBV), a partially double-stranded hepatotropic DNA virus, is the etiological agent of acute and chronic hepatitis B in humans. Chronic HBV infection prevalence among the Italian population is estimated to be 0.7% (Istituto superiore di Sanità, 2022; Sheena et al., 2022). Coinfection with Human Immunodeficiency Virus (HIV) and Hepatitis C Virus (HCV) is common among HBV-infected individuals due to shared routes of transmission, and it is often associated with worse immunological control and advanced liver disease (Puglia et al., 2016; Zhou et al., 2020). After acute HBV infection the outcome varies widely between subjects, and encompasses resolution, asymptomatic carriage and chronic active hepatitis. The most important determinants of chronicity are the immune status of the host and the age at the time of infection. Diagnosis of HBV infection status requires a plethora of serological and virological tests (Lampertico et al., 2017). Occult HBV infection (OBI) is defined as the presence of replication-competent HBV DNA (i.e. episomal HBV covalently closed circular DNA [cccDNA]) in the liver and/or HBV DNA in the blood of people who test negative for hepatitis B surface antigen (HBsAg) by currently available assays. Detection of serum anti-HBc is often used as a surrogate for the diagnosis of OBI (Raimondo et al., 2019). However, its precise prevalence is yet to be clearly defined (Im et al., 2022). Under long-term immunosuppression, i.e., during chemotherapy, hematological malignancies, steroid therapy or Acute immunodeficiency syndrome (AIDS), HBV reactivation might occur as consequence of the presence of replication-competent HBV DNA in the liver’s hepatocytes. Reactivation is characterized by a sudden increase in serum HBV DNA levels, HBsAg reappearance and is often associated with a hepatitis flare (Perrillo et al., 2015). HBV infection prevalence among HIV-infected patients is now decreasing thanks to vaccination campaigns and the new antiretroviral regimens (ART) allow good immunological status in people living with HIV (PLWH): drugs such as tenofovir, which proved to be a potent HBV inhibitor, are often part of them: thanks to all these factors HBV-related chronic disease generally is not an issue and OBI reactivation is now rather infrequent among PLWH (Huang and Núñez, 2015; Soriano et al., 2019). HCV infection can be associated with HBV, and OBI is particularly common among patients affected by chronic hepatitis C. This may be related to HCV dominance over HBV, which determines HBsAg loss (Witt et al., 2013). HBV reactivation has been reported in patients starting HCV therapies who are not on active HBV agents. Typically, HBV reactivation occurs 4 to 8 weeks after direct-acting antivirals (DAAs) initiation. Because of that, International Guidelines recommend that all patients initiating DAAs be screened for HBV, and HBsAg negative, anti-HBc positive patients be monitored for HBV reactivation (Chang et al., 2018). Here we present a case of OBI reactivation in an HIV/HCV co-infected patient with severe immunodeficiency after self-suspension of ART and HCV eradication, following reintroduction of antiretroviral therapy.

## Case description

A 64-year-old Caucasian man with an history of drug abuse came to the attention of our Infectious Diseases Unit solely in 1991 for HIV infection and non-A non-B hepatitis starting an irregular follow-up. HIV infection was first diagnosed in 1984. ART was not immediately started due to his good immunological status (CD4 cell count: 976 cell/mm3) and the ongoing use of recreational drugs. During the monitoring laboratory tests, isolated anti-HBc seropositivity was identified and chronic HCV hepatitis was diagnosed. In 1997, he initiated a drug rehabilitation program and, due to the worsening of his immunological status (CD4 cell count: 198 cell/mm3), an antiretroviral stavudine/lamivudine-containing regimen was promptly started. Although at first an irregular follow-up was started due to the poor adherence, the patient begun to regularly follow the scheduled visits thanks to the rehabilitation program. Several antiretroviral regimens were switched according to the over-time changing guidelines always reaching an optimal and maintained viro-immunological response. As regards his HCV infection, in 2016, he started a ledipavir/sofosvubir/ribavirin combination therapy for 24 weeks reaching sustained viral response (SVR). No changes were reported in the HBV serology with both anti-HBs and HBsAg persistently negative as shown in **Table 1**. Unfortunately, in early 2018, the patient was lost to follow up.

**Table 1:** HIV and HBV viral and immunological markers before the loss of care and after the re-engagement.

Date	CD4 (cell/mm3)	CD8 (cell/mm3)	% CD4	% CD8	CD4/CD8	HIV-RNA (cp/mL)	HBV-DNA (UI/mL)	AST (U/L)	ALT (U/L)	HBsAg quant (UI/L)	HBsAg qual
01/17	1074	1799	25	41,9	0,6	< 20	NA	19	21	NA	negative
04/17	1234	1953	26,3	41,6	0,6	< 20	NA	20	18	NA	negative
01/18	1154	1807	26,8	42	0,6	< 20	NA	18	22	NA	NA
06/21	104	1045	8	79,9	0,1	73142	NA	40	21	NA	positive
09/21	186	2241	6,7	80,4	0,08	< 20	63200	57	53	NA	NA
03/22	184	1585	8,1	69,3	0,12	< 20	NA	105	143	1589	positive
10/22	118	1035	7,4	64,3	0,11	< 20	< 10	27	16	NA	NA
12/22	195	1411	9,4	67,7	0,14	< 20	< 10	22	11	NA	negative

NA, Not Applicable; Quant, Quantitative; Qual, Qualitative.

In June 2021, he resumed the follow-up visits showing an alarming viro-immunological profile: high levels of HIV-RNA were detectable and the CD4 cell count was less than 200 cells/mm3. ART and the opportunistic infection prophylaxis were promptly started. Few months later, although HIV-RNA went back to be undetectable together with an improved immunological profile, HBsAg turned positive with a detectable and high-level HBV DNA viraemia associated with an increase in transaminases. A reactive switch to an integrase inhibitor (INI)-based regimen containing tenofovir (emtricitabine/tenofovir-alafenamide/bictegravir) was started. At the end of 2022, hepatic indexes normalized, and HBV-DNA was stably undetectable.

## Discussion

The case of OBI reactivation in a HIV/HCV co-infected patient presents several aspects that deserve to be discussed. A 64-year-old patient with HIV/HCV/OBI and cirrhosis followed for 25 years at our clinic was lost to follow-up about 19 months after DAAs treatment. Blood tests performed before loss of care showed an optimal immunological status with suppressed HIV viral load, isolated anti-HBc seropositivity and a sustained viral response (SVR) for HCV. At re-engagement (42 months after the last visit) blood tests showed high replication of plasmatic HIV-RNA (73142 cp/mL) along with severe immunosuppression and normal levels of liver enzymes. Three months after ART reintroduction, both CD4 and CD8 counts increased, and transaminases (ALT/AST) were slightly elevated.

Currently, occult HBV infection management and HIV management guidelines (EACS) indicate close monitoring for OBI/HIV coinfection, especially if the HIV drug regimen of choice does not include drugs with activity against both HIV and HBV (European AIDS Clinical Society, 2021). Moreover, particular attention is required for patients switching to a drug sparing regimen that does not involve either lamivudine or tenofovir. Rouphael et al, described one of the largest series of HBV reactivation in PLWH, estimating an incidence ratio of 0.019 cases per 100 person-year (Rouphael et al., 2007). Mican et al. more recently described an episode of HBV reactivation associated with a severe hepatitis flare in an HIV-infected patient with a resolved hepatitis B, three months after removing TDF from his regimen, as part of a simplification strategy (Mican et al., 2021). HBV reactivation mostly occurs in the setting of worsening CD4 cell counts or with a CD4 count <100 cells/μL. Also, HBV-related hepatic flare may occur in HIV-infected patients during an immune reconstitution syndrome (IRIS) after the initiation of an active antiretroviral treatment (Iannetta et al., 2022). This shows how the balance between the virus and the host immune system is quite a dynamic process that may significantly impact the course of the disease.

The natural history of OBI and the mechanism involved in HBV reactivation are only partially understood (Raimondo et al., 2019). OBI status is associated with an anti-HBV immune response that can exert a strong suppression over viral replication. OBI reactivation depends both on the specific virus characteristics and on several host factors such as older age, underlying diseases and the use of immunomodulant therapies (Shih and Chen, 2021).

HCV/HBV coinfection deserves further considerations. Co-transfection studies revealed that the HCV core protein may be involved in the inhibition of HBV replication (Shih et al., 1993). Therefore, quick, and effective DAA suppression of HCV replication could result in increased HBV viral replication or, in case of an OBI, even reactivation. A recent review and meta-analysis estimated a minimal risk of HBV reactivation and seroreversion in patients with OBI. Nonetheless, follow-up of HBV serological and virological markers during 24 weeks after treatment with DAA is generally advised (Mücke et al., 2018). Regarding our patient, however, the time elapsed between DAA therapy and OBI reactivation allows us to exclude HCV eradication as a possible cause of HBV reactivation.

Regarding HIV/HBV coinfection, OBI reactivation is rather rare nowadays due to the widespread use of effective antiretroviral regimens that include anti-HBV agents and suppress HBV replication (Soriano et al., 2019). Nonetheless, HBV reactivation might occur in patients with HIV/HBV coinfection when antiretroviral regimens are modified and HBV active drugs are withdrawn (i.e., dual therapy with tenofovir or lamivudine sparing regimens) (Raimondo et al., 2019).

Several hypotheses have been formulated to explain OBI reactivation and HBV-related disease in our patient. Firstly, the failure of immune surveillance and the following immune reconstitution-induced inflammatory syndrome (IRIS) after ART reintroduction. Secondly, reinfection cannot be excluded but is rather unlikely because immunity to HBV is developed against several epitopes and should be protective against other possible HBV subtype infections (Wang et al., 2021).

The outcomes of HBV infection depend on ever-evolving and dynamic interaction and balance between HBV replication and the host immune response, which involves humoral and cellular immunity with effectors T cells (CD4 cells, CD8 cells and T-Reg), B-cells and neutralizing antibodies (Gherlan, 2022).

CD8 cells have a pivotal role in the viral clearance and pathogenesis during acute HBV infection as well as the chronic phase because they recognize and eliminate infected hepatocytes (Thimme et al., 2003). HBV-specific CD8 functional exhaustion or clonal deletion are responsible for viral persistence during the chronic infection (Heim et al., 2019; Zhang et al., 2022). CD4 cells also play an important role in the immune response against HBV by activating innate immune cells, priming CD8 cells, maintaining CD8 cell effector function aimed to cell-mediated HBV clearance and inducing B cells to produce antibodies (Yang et al., 2010; Buschow and Jansen, 2021). An impaired HBV-specific CD4 cell response has been associated with HBV persistence even in presence of HBV-specific CD8 cells in HBV/HCV co-infected patients (Urbani et al., 2005).

Wang et al. described a positive relation between frequency of HBV-specific CD4 cells (mainly HBV core-specific TNF-α producing CD4 cells), hepatitis B flares and liver damage, indicating robust on-going T cell responses in patients with HBV chronic infection (Wang et al., 2021). A further differentiation of those HBV-specific TNF-α producing CD4 cells into IFN-γ producing CD4 cells favors HBV viral clearance.

The incidence of HBV hepatitis flare in rheumatic patients with OBI receiving rituximab is rather rare (3.4 per 1,000 person-years) and lower than the incidence of HBV reactivation confirming that the cooperation among all immune cells is necessary to effective immune response after HBV reactivation (Lan et al., 2022).

B-cell depleting agents such as rituximab or ofatumumab are associated with the highest risk for HBV reactivation (>10%). All patients exposed to HBV, regardless of the HBsAg status, should start antiviral prophylaxis before initiating B-cell depleting therapies (Koffas et al., 2018). In HBsAg-negative and anti-HBc positive subjects with moderate (< 10%) or low (< 1%) risk of HBV reactivation, pre-emptive therapy, instead of prophylactic therapy, is generally recommended. It should be noted, however, that specific measures for PLWH with the same serological pattern are not provided by International Guidelines. Pre-emptive therapy consists in starting antiviral therapy in case of detectable HBV-DNA or HBsAg sero-reversion during follow up (Koffas et al., 2018; Papatheodoridis et al., 2022).

At re-engagement, our patient showed no signs of a hepatitis flare, probably due to his severe immunodeficiency and low CD4 cells count. It was only when CD4 cells improved, following the antiretroviral therapy reintroduction, that a slight elevation in transaminases was described, concomitantly to a significant increase of CD8 cells. This was suggestive of IRIS occurring after ART reinstatement. It has hypothesized that IRIS could contribute to HBsAg loss, especially when liver inflammation ensues early after ART introduction (Yoshikawa et al., 2021). The recovery of HBV-specific cytotoxic CD8 cells, has been speculated as the cause of this phenomena (Yoshikawa et al., 2021; Iannetta et al., 2022). Ultimately, the eminent hepatic flare was likely due to IRIS in our case because IRIS diagnostic criteria were present (Brust et al., 2021). Also, the hepatitis flare was associated with seroreversion for HBsAg. Fortunately, the prompt initiation of specific antiviral treatment with tenofovir alafenamide as part of the ART regimen, quickly resolved the hepatic flare and the plasmatic HBV-DNA became negative once again.

## Conclusion

Hepatic damage and HBV clearance are mediated by the host immune response, which can suppress viral replication to minimal levels, achieving control of the infection and, ultimately, the OBI status. However, when multiple factors associated with a low chance of OBI reactivation are concomitantly present, the risk of this complication may exponentially increase. The aim of this case report was to bring to the attention of physicians that, although OBI reactivation is a rather rare occurrence, even amongst PLWH, its potential consequences compel to a high alertness on the matter. Therefore, especially in patients with an impaired immune system and on a tenofovir or lamivudine-sparing regimen, HBV serological and virological markers should always be strictly monitored, even in the absence of a hepatitis flare.

## Data availability statement

The original contributions presented in the study are included in the article/supplementary material. Further inquiries can be directed to the corresponding author.

## Ethics statement

Written informed consent was obtained from the individual(s) for the publication of any potentially identifiable images or data included in this article.

## Author contributions

Conceptualization: EQ-R. Writing–original draft preparation: SZ, AC, MD, CC, SS, GT, FC, and EQ-R. Methodology: SZ, and EQ-R. Data curation: SZ, AC, MD, CC, SS, GT, FC, and EQ-R. Writing—review and editing: SZ, AC, MD, CC, SS, GT, FC, and EQ-R. Visualization: SZ, AC, MD, CC, SS, GT, FC, and EQ-R. Supervision: SZ, and EQ-R. All authors have read and agreed to the submitted. All authors contributed to the article and approved the submitted version.
